# Large-Scale Bioinformatics Analysis of *Bacillus* Genomes Uncovers Conserved Roles of Natural Products in Bacterial Physiology

**DOI:** 10.1128/mSystems.00040-17

**Published:** 2017-11-14

**Authors:** Kirk J. Grubbs, Rachel M. Bleich, Kevin C. Santa Maria, Scott E. Allen, Sherif Farag, AgBiome Team, Elizabeth A. Shank, Albert A. Bowers

**Affiliations:** a Department of Biology, University of North Carolina, Chapel Hill, North Carolina, USA; b Eshelman School of Pharmacy, University of North Carolina, Chapel Hill, North Carolina, USA; c Department of Chemistry, University of North Carolina, Chapel Hill, North Carolina, USA; d AgBiome, Research Triangle Park, North Carolina, USA; e Department of Microbiology and Immunology, University of North Carolina, Chapel Hill, North Carolina, USA; University of Pennsylvania

**Keywords:** *Bacillus*, bioinformatics, natural products, nonribosomal peptide synthetase, polyketides, secondary metabolism, specialized metabolites, sporulation, thiopeptides

## Abstract

Bacteria possess an amazing capacity to synthesize a diverse range of structurally complex, bioactive natural products known as specialized (or secondary) metabolites. Many of these specialized metabolites are used as clinical therapeutics, while others have important ecological roles in microbial communities. The biosynthetic gene clusters (BGCs) that generate these metabolites can be identified in bacterial genome sequences using their highly conserved genetic features. We analyzed an unprecedented 1,566 bacterial genomes from *Bacillus* species and identified nearly 20,000 BGCs. By comparing these BGCs to one another as well as a curated set of known specialized metabolite BGCs, we discovered that the majority of *Bacillus* natural products are comprised of a small set of highly conserved, well-distributed, known natural product compounds. Most of these metabolites have important roles influencing the physiology and development of *Bacillus* species. We identified, in addition to these characterized compounds, many unique, weakly conserved BGCs scattered across the genus that are predicted to encode unknown natural products. Many of these “singleton” BGCs appear to have been acquired via horizontal gene transfer. Based on this large-scale characterization of metabolite production in the *Bacilli*, we go on to connect the alkylpyrones, natural products that are highly conserved but previously biologically uncharacterized, to a role in *Bacillus* physiology: inhibiting spore development.

**IMPORTANCE** *Bacilli* are capable of producing a diverse array of specialized metabolites, many of which have gained attention for their roles as signals that affect bacterial physiology and development. Up to this point, however, the *Bacillus* genus’s metabolic capacity has been underexplored. We undertook a deep genomic analysis of 1,566 *Bacillus* genomes to understand the full spectrum of metabolites that this bacterial group can make. We discovered that the majority of the specialized metabolites produced by *Bacillus* species are highly conserved, known compounds with important signaling roles in the physiology and development of this bacterium. Additionally, there is significant unique biosynthetic machinery distributed across the genus that might lead to new, unknown metabolites with diverse biological functions. Inspired by the findings of our genomic analysis, we speculate that the highly conserved alkylpyrones might have an important biological activity within this genus. We go on to validate this prediction by demonstrating that these natural products are developmental signals in *Bacillus* and act by inhibiting sporulation.

## Abstract (precis)

*Bacilli* are capable of producing a diverse array of specialized metabolites, many of which have gained attention for their roles as signals that affect bacterial physiology and development. Up to this point, however, the *Bacillus* genus’s metabolic capacity has been underexplored. We undertook a deep genomic analysis of 1,566 *Bacillus* genomes to understand the full spectrum of metabolites that this bacterial group can make. We discovered that the majority of the specialized metabolites produced by *Bacillus* species are highly conserved, known compounds with important signaling roles in the physiology and development of this bacterium. Additionally, there is significant unique biosynthetic machinery distributed across the genus that might lead to new, unknown metabolites with diverse biological functions. Inspired by the findings of our genomic analysis, we speculate that the highly conserved alkylpyrones might have an important biological activity within this genus. We go on to validate this prediction by demonstrating that these natural products are developmental signals in *Bacillus* and act by inhibiting sporulation.

## INTRODUCTION

Bacteria devote large portions of their genomes to the genes necessary for natural product biosynthesis. These specialized metabolites (formally called secondary metabolites) have historically been crucial to the development of clinically relevant antibiotics. More recently, they have garnered attention as intraspecies and interspecies signals that affect bacterial physiology and development (1–5). In light of the important therapeutic and ecological roles that specialized bacterial metabolites play, there is broad interest in identifying the entire repertoire of metabolites that bacteria are capable of generating, as well as in identifying their biological functions.

Most specialized metabolites are structurally complex biomolecules that fall into distinct structural classes synthesized by conserved classes of biosynthetic metabolites. This association between metabolite class and a metabolite’s gene signature allows genome sequences to be searched to identify predicted biosynthetic gene clusters (BGCs). Such genome mining has dramatically increased the rate of discovery of specialized metabolites compared to those of traditional methods, which rely on the isolation of individual compounds (6, 7). A number of large-scale genome-mining projects have focused on defining the metabolite-encoding potential of *Actinobacteria*, a bacterial group known to be prolific producers of natural products and the taxon that has supplied the majority of clinically deployed antibiotics (8). In contrast, we focus here on the Gram-positive *Bacilli*. *Bacillus* is a metabolically underexplored genus that nevertheless possesses substantial biosynthetic potential.

*Bacillus* species are omnipresent in soils and in the plant rhizosphere, and many have also been harnessed for industrial and agricultural applications. Some species are considered safe biological agents and are used as plant-growth-promoting bacteria or food additives, while others are pathogens (9, 10). In addition to having agricultural importance and ecological diversity, the specialized metabolites of *Bacilli* play significant roles in the interactions and life cycles of these bacteria as both intra- and interspecies signals. Many specialized *Bacillus* metabolites coordinate cellular differentiation (11, 12). For example, the nonribosomally produced lipopeptide surfactin is important for development of multicellularity in *Bacillus subtilis*, acting as a quorum-sensing-like signal that stimulates biofilm production (13, 14). Surfactin also acts as an interspecies signal, inhibiting aerial hyphal formation in *Streptomyces coelicolor* (15), and as an antibiotic (it is one of more than two dozen antibiotics that have been isolated from strains of *Bacillus subtilis*). Bacillaene, a hybrid nonribosomal peptide and polyketide, is also involved in interspecies interactions; bacillaene inhibits prodiginine production in *Streptomyces* species (16) and protects *B. subtilis* against predation by *Myxococcus xanthus* (17). More recently, a group of ribosomally synthesized and posttranslationally modified peptides (RiPPs) produced by *B. cereus* known as thiocillins have been shown to stimulate *B. subtilis* to produce biofilm in a manner that is independent of antibiotic activity (18, 19). Thus, these specialized metabolites are an important means by which *Bacilli* interact with themselves and their surroundings.

Here we deploy intensive bioinformatics to comprehensively survey the breadth and diversity of the chemical ecology encoded within *Bacillus* genomes. Although previous efforts have sought to assess the metabolic diversity of this genus (20), our analysis exploits new and significantly larger data sets to measure the conservation of BGCs across *Bacilli* and to distinguish secondary metabolites involved in their life cycles and social interactions. Our analysis agrees with the concept that metabolites associated with highly conserved biosynthetic genes are likely to play important roles regulating *Bacillus* physiology and development. As evidence of this trend, we isolate a highly conserved group of alkylpyrones and elucidate their role as chemical messengers that regulate sporulation in *B. subtilis*. Additionally, a large number of *Bacilli* possess rare BGCs that encode unknown metabolites that have been acquired predominantly through horizontal transfer. Our results provide insights into key molecular characteristics of the chemical ecology of *Bacilli* and provide a strong foundation for future assessments of the biosynthetic capabilities of this genus.

## RESULTS

### Genome database and analysis.

One of the distinctive features of our analysis is the large number of sequences from a single genus that we examined. We analyzed the 221 *Bacillus* genomes that were publicly available at the time that we began this work, as well as 1,345 proprietary *Bacillus* genomes from AgBiome. By comparison, we have evaluated nearly five times more genomes than the next-most-extensive published analyses (20) and significantly more than other similar analyses of other bacterial genera (8). Additionally, the genomes that we analyzed are being made newly available to the public together with this analysis, greatly enhancing the number of *Bacillus* genome sequences available to the research community.

In order to designate species names with the sequences in our data set, we used Barrnap to identify 16S and 23S ribosomal gene sequences in each genome and used the predicted sequences in a BLAST search against the Genomic Reference Sequence Database (GenomicRefSeq). To streamline the presentation of our results, we have assembled the *Bacilli* in our data set into groups containing phylogenetically related species: the *cereus*, *subtilis*, *flexus*, *coagulans*, and “other” groups (see Fig. 2A for a summary of all species, group members, and numbers of strains per species). While members of both of the best-characterized *Bacillus* clades (the *subtilis* and *cereus* groups) live in the soil, they occupy distinct niches within that habitat: the *subtilis* group is associated primarily with plants, while the *cereus* group is associated primarily with insects.

**FIG 1:**
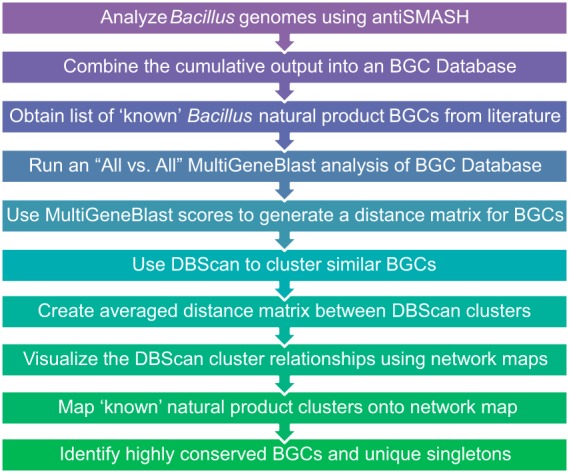
Workflow for bioinformatics calculations.

To evaluate the biosynthetic potential of these *Bacilli*, we developed the analysis pipeline outlined in Fig. 1. Our workflow integrates several methods to effectively analyze and visualize the biosynthetic capabilities of *Bacilli* and draw meaningful comparisons. We began by using antiSMASH (21) to analyze the biosynthetic capacities of our 1,566 genomes. The cumulative output from antiSMASH was 19,962 biosynthetic gene clusters (BGCs), which included nonribosomal peptide synthetases (NRPSs), polyketide synthases (PKSs), bacteriocins, thiopeptides, lanthipeptides, terpenes, ectoines, phosphonates, siderophores, and hybrid/nontraditional BGCs. Representatives of 23 characterized *Bacillus* BGCs (where a natural product is associated with a BGC [see Table S1 in the supplemental material]) were combined with these predicted BGCs for a 19,985-member MultiGeneBlast (MGB) library. The BGCs were analyzed using MultiGeneBlast (22), a program that does an all-by-all comparison and calculates an MGB score for each BGC pair based on the synteny of the genes within each BGC and their BLAST scores. MGB scores were normalized and used as input for density-based spatial clustering of applications with noise (DBSCAN) (23) to cluster similar BGCs. Parameters selected for DBSCAN clustering (see Materials and Methods) were conservative, such that only highly related BGCs were placed in the same DBSCAN cluster. In some cases, this led to multiple DBSCAN clusters being generated for related molecular families. Although the stringency in our cutoffs may be a potential source of error, it should result in only significant relationships being exposed. The quality of the DBSCAN clustering was confirmed through a silhouette coefficient calculation (24) for each class of metabolites (Table S2). Using the averaged MGB scores, we then generated a distance matrix to describe the relatedness of each DBSCAN cluster to all other DBSCAN clusters in our MGB database. We sorted the resulting DBSCAN clusters by the class of natural product that they contained (e.g., NRPS, PKS, etc.) based on antiSMASH’s assignments. We then used Cytoscape (25) to build network maps that allowed the relationships between the DBSCAN clusters to be visualized. These network maps provide an overview of the overall placement in clades of specialized metabolites within the *Bacilli*. By mapping the characterized, known BGCs onto the network maps, the vast unknown diversity of specialized *Bacillus* metabolites was revealed, as described below.

10.1128/mSystems.00040-17.7 TABLE S1  *Bacillus* compounds with known BGCs. Download TABLE S1, EPS file, 1.2 MB. Copyright © 2017 Grubbs et al. 2017 Grubbs et al. This content is distributed under the terms of the Creative Commons Attribution 4.0 International license.

10.1128/mSystems.00040-17.8 TABLE S2  Overview of *Bacillus* species and BGC diversity and DBSCAN-optimized parameters with silhouette coefficient scores. Download TABLE S2, EPS file, 0.7 MB. Copyright © 2017 Grubbs et al. 2017 Grubbs et al. This content is distributed under the terms of the Creative Commons Attribution 4.0 International license.

In addition to performing these analyses, we used Alien_hunter (26) to predict DNA regions that are likely to have been obtained via horizontal gene transfer (HGT). We overlaid these results with those obtained from antiSMASH to determine how much of each BGC was obtained via HGT. antiSMASH is highly conservative in defining the boundaries of BGCs (so as to avoid excluding uncharacterized tailoring enzymes), which frequently leads to nonbiosynthetic regions of the genome being included in antiSMASH’s output of BGCs. Because of this, we used a cutoff of 75% overlap for designating BGCs as predicted to be obtained via HGT.

To verify our bioinformatics methods, we examined a subset of bacterial strains predicted to produce known metabolites. For structures of known *Bacillus* metabolites, see Fig. S1. Using established procedures, we used high-resolution liquid chromatography-mass spectrometry (LCMS) to survey the cell surface extracts of strains predicted to produce kurstakins (2 public and 36 AgBiome strains) and those predicted to produce bacillamide (3 public and 16 AgBiome strains). We found masses corresponding to kurstakin and bacillamide production in 79% and 58% of the strains predicted to produce these metabolites, respectively (Fig. 3C and Fig. S2). We speculate that other strains (those predicted to make these compounds but which have not been detected to make them) may contain “silent” BGCs, where the metabolites are not expressed under laboratory growth conditions (27). This analysis confirms the effectiveness of our bioinformatics predictions and underscores the prevalence of these compounds in *Bacillus* strains.

10.1128/mSystems.00040-17.1 FIG S1  Structures of known *Bacillus* metabolites. Download FIG S1, EPS file, 1.7 MB. Copyright © 2017 Grubbs et al. 2017 Grubbs et al. This content is distributed under the terms of the Creative Commons Attribution 4.0 International license.

10.1128/mSystems.00040-17.2 FIG S2  LCMS distributions of kurstakins and bacillamides in predicted strains. The area under the extracted ion chromatogram (EIC) gives the relative integration value for each extracted ion peak. Download FIG S2, EPS file, 1.2 MB. Copyright © 2017 Grubbs et al. 2017 Grubbs et al. This content is distributed under the terms of the Creative Commons Attribution 4.0 International license.

### General insights.

**FIG 2:**
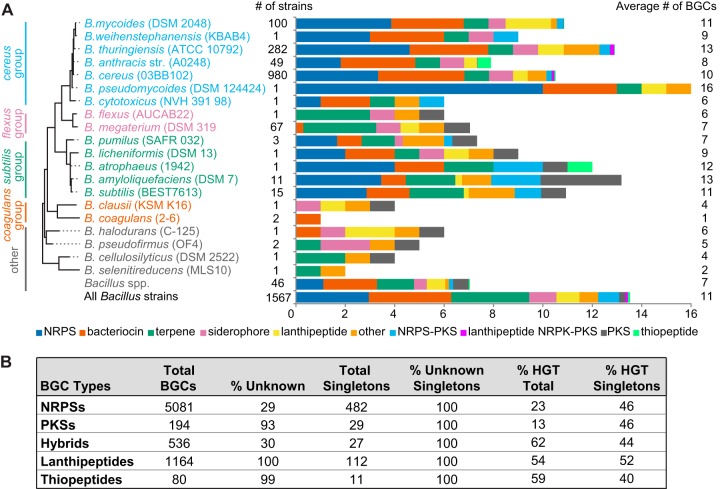
Overview of *Bacillus* species and BGC diversity. (A) Phylogenetic and chemical diversity of *Bacillus* species. A phylogenetic tree shows representative strains and colored boxes that designate the species groups used throughout. Types and abundances of BGCs found within each species are shown in a graph, with total numbers of strains on the left and average numbers of BGCs per strain for each species on the right. (B) Distribution of BGC totals and singletons by type along with overall percentages of BGCs above a horizontal gene transfer score of 75.

Overall, we find that the *Bacilli* are relatively homogeneous in their metabolite class distributions, particularly within each phylogenetic clade. For instance, species from the *cereus* and *subtilis* groups have large numbers of NRPSs and bacteriocins, while the *flexus* and “other” groups are highly enriched in terpenes (Fig. 2A). *Bacilli* average 11 BGCs per strain, but this number can vary between 1 and 16, depending on the strain (Fig. 2A). Note that a large proportion of the strains that we have analyzed are in the *cereus* group; this bias also afforded us the chance to more deeply sample this important portion of the *Bacillus* taxon and gain further insight into metabolite conservation within a single bacterial species.

**FIG 3:**
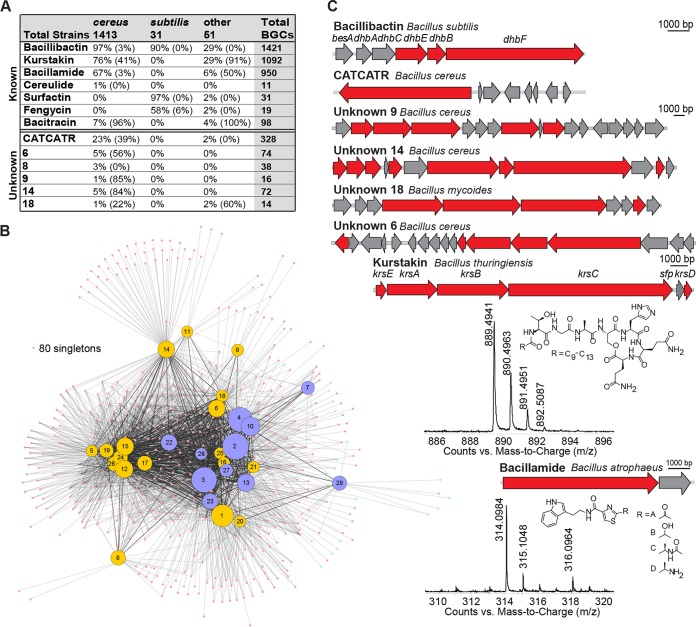
NRPS complexity. (A) Shown is a table with a breakdown of the percentages of strains containing each type of NRPS by species group followed by the percentages of BGCs above a 75 HGT score in parentheses. (B) The network map visually shows the known (purple) and unknown (yellow) NRPS clusters, with the cluster number at each node. The number of singletons is noted. (C) Representative NRPS BGCs are indicated, with NRPS genes in red. The structures and compound production of bacillamide and kurstakin are highlighted with their BGCs.

**FIG 4:**
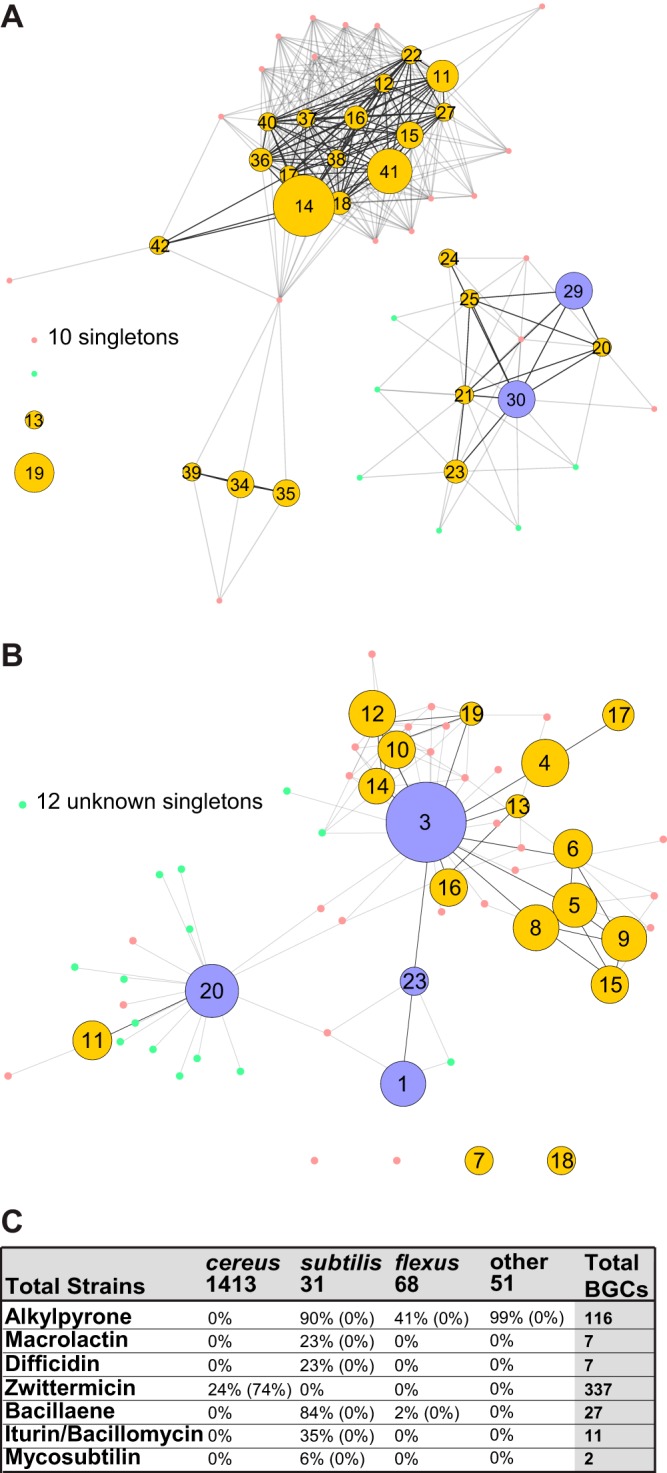
PKS distribution. (A) Network map for known (purple) and unknown (yellow) PKS clusters, with the cluster number at each node and the number of singletons listed. (B) Network map for known (purple) and unknown (yellow) hybrid NRPS-PKS clusters, with the cluster number at each node. (C) Distribution of the most-abundant PKS BGCs between species groups showing the percentages of strains containing that BGC with the percentages of strains above a 75 HGT score in parentheses.

The distributions of known and unknown BGCs vary dramatically across the different specialized metabolite classes (Fig. 2B). NRPSs are the most abundant class of specialized metabolite within our data set, comprising 5,081 BGC representatives (Fig. 2B). However, NRPSs are conspicuously absent from the *flexus* and *coagulans* groups (Fig. 3A). The majority of the NRPS BGCs cluster with known compounds; only 1,140 BGCs, roughly 22% of the predicted NRPSs, appear to be responsible for new or novel compounds. Unlike with these well-described NRPS BGCs, the majority of PKS, lanthipeptide, and thiopeptide BGCs are unknown (Fig. 2B). Overall, there are far fewer PKS BGCs in *Bacilli* than NRPS BGCs, and PKSs are present primarily in the *subtilis* group strains (Fig. 4C). Type III PKSs dominate, while the remaining BGCs are mostly type I trans-AT PKSs (trans-AT PKSs do not have the acetyltransferase domain encoded within the PKS modules within the BGC, but instead a separate protein encoded in the BGC will act in *trans*); with only a few exceptions, type II PKS BGCs appear to be predominantly absent from *Bacillus*. We also observed that hybrid NRPS-PKS enzymes are more abundant in *Bacilli* than PKS BGCs are (Fig. 2B). Zwittermicin, a type I PKS-NRPS hybrid, accounts for the majority of all hybrid BGCs; the remaining hybrid systems predominantly use the trans-AT PKS-NRPS machinery. Like the NRPSs, the majority of hybrid NRPS-PKS BGCs from *Bacilli* are predicted to produce known compounds, but there is a small cohort (133 BGCs) that can be attributed to unknown and potentially new or novel compounds. Lastly, canonical RiPPs (e.g., lanthipeptides and thiopeptides) have a much smaller percentage of known members. We identify only 5 known compounds from the 1,164 detected lanthipeptide BGCs, while the thiopeptides exhibit only 1 known representative among the 80 identified BGCs.

Thus, depending on the metabolite class, many of the identified BGCs in our data set appear to be conserved (i.e., present in a significant proportion of strains) among the *Bacilli*. Most of the NRPS BGCs are highly conserved across different *Bacillus* species, indicating their importance to the genus. Correspondingly, these NRPSs show little evidence of HGT. Although the *Bacillus* PKSs are mostly attributed to unknown natural products (with the exception of zwittermicin, the only PKS identified in the *cereus* group), the few PKS BGCs that were found appear to be core constituents of the genus and show little evidence of HGT. Remarkably, essentially all of the unique singletons in our data set (661 total BGCs) are attributed to unknown compounds, irrespective of their metabolite class. Many of these poorly conserved, rare BGCs that are scattered throughout the *Bacillus* genomes appear to have been acquired by HGT. Below, we discuss the known and highly conserved BGCs, the unknown and highly conserved BGCs, and a few compelling examples of poorly conserved BGCs.

### Known, highly conserved BGCs.

Many of the highly conserved BGCs in our data set encode NRPSs; a few additional hybrid NRPS PKSs, such as zwittermicin and bacillaene, are also well conserved. Although several of these compounds have already been linked to important signaling roles in *Bacilli*, the native biological activities of many of them remain poorly understood.

The most abundant NRPS BGC in the data set by far is the biosynthetic pathway for the siderophore bacillibactin, the primary iron scavenger for most *Bacillus* species (Fig. 3B, cluster 2) (28). Bacillibactin was identified in 90.9% of the public strains (200 genomes) as well as 90.7% of the AgBiome strains (1,220 genomes). Of the remaining 146 strains that lack bacillibactin, 130 were found to contain putative petrobactin BGCs; petrobactin is the primary siderophore in *Bacillus anthracis* (29). With regard to the remaining 16 strains, we hypothesize that either (i) they generate alternative, as-yet-unknown siderophores or (ii) they do not make their own siderophores but instead exclusively “pirate” siderophores produced by other bacteria (30).

The second-most-common group of NRPS BGCs was the kurstakin synthetases. The kurstakins are cyclic lipopeptides that control swarming and biofilm formation in *Bacillus thuringiensis* (31). BGCs for the kurstakins were identified in 42.5% of the public strains (94 genomes) and 50% of the AgBiome strains (998 genomes); these BGCs are found only in the *cereus* group and in one strain from the “other” group (Fig. 3B, clusters 3 and 13). In addition to the high conservation of the six kurstakin biosynthetic genes, there is high conservation within the amino acids incorporated by these synthetases in our data set strains; variation was typically limited to the sixth amino acid.

Bacillamide biosynthetic genes were unexpectedly common in the data set (Fig. 3B, cluster 4). To date, the algicidal bacillamides have been isolated only from marine *Bacillus* strains (32–34). However, these BGCs were present in 45.7% of the public strains (101 genomes) and 42.8% of the AgBiome strains (849 genomes). They were observed in *B. thuringiensis*, *B. cereus*, *B. anthracis*, *Bacillus mycoides*, and several of the “other” strains. Other than their algicidal properties and activities against one strain of cyanobacteria, little is known about whether bacillamides play signaling roles within the *Bacilli*; their prominence indicates that they may serve an important biological function in the *cereus* group in particular.

Zwittermicin (Fig. 4B, cluster 3) is an aminopolyol with broad-spectrum antibiotic activity that was first isolated from *B. cereus* (35). This type I PKS-NRPS hybrid BGC has been identified in a number of *Bacillus* strains since then, and it appears in 8.1% of the public strains (18 genomes) and 23.7% of the AgBiome strains (319 genomes) (Fig. 4C). Although the zwittermicin BGC is common within these *Bacillus* strains, our data indicate that it was likely acquired via HGT (Fig. 4C). Zwittermicin has been noted for its synergistic activity with other microbial metabolites. For instance, it synergizes with kanosamine to inhibit the growth of *Escherichia coli* and oomycetes; it was later discovered that in some strains, the kanosamine biosynthesis genes are found within the zwittermicin BGC (36, 37). Our data set reveals that zwittermicin is also frequently associated with other BGCs, including three separate class II lanthipeptides and a group of NRPS BGCs with a conserved CATCATR modularity. Although the products of these BGCs are unknown, their consistent integration into the zwittermicin cluster suggests a potential synergistic or regulatory role.

There are a number of other notable, highly conserved, and known NRPS-derived BGCs within our data set. Specifically, bacillaene, a hybrid NRPS PKS (Fig. 4B, cluster 20), and surfactin and fengycin, NRPSs (Fig. 3B, clusters 29 and 27, respectively), are common among *subtilis* group members, while bacitracin (Fig. 3B, clusters 22 and 23) and cereulide (Fig. 3B, cluster 28) are predominantly found within *cereus* group members (Fig. 3A). Many of the best-known and best-characterized *Bacillus* NRPS metabolites, such as bacillibactin, bacillamide, cereulide, surfactin, and fengycin, exhibit a very low likelihood of HGT, at least within the *cereus* group (Fig. 3A). In contrast, two other known compounds (kurstakin and bacitracin) showed a high likelihood of having been acquired by HGT (Fig. 3A).

### Unknown, highly conserved BGCs.

A number of well-conserved BGCs did not readily connect with known natural products in our analysis, suggesting that they may synthesize new or novel compounds. Two such BGCs were particularly prevalent: (i) a group of NRPS BGCs with a conserved CATCATR architecture and (ii) a type III PKS which had previously been hypothesized to make a series of alkylpyrones in *B. subtilis*.

The stringent parameters used in our analysis meant that the CATCATR BGC was split into a number of smaller DBSCAN clusters (Fig. 3B, clusters 5, 11, 12, 15, 17, 19, 24, and 26; cluster 11 groups separately from the others due to its unique genomic context). Although these BGCs were found in 5.4% of public strains (12 genomes) and 24.4% of AgBiome strains (316 genomes), this BGC remains largely uncharacterized. Its NPRS gene architecture suggests that it generates a modified dipeptide which may be structurally related to a series of diketopiperazines recently found to regulate virulence in *Staphylococcus aureus* (38, 39). Notably, these BGCs show a high likelihood of having been acquired via HGT (Fig. 3A).

Even though PKSs are relatively rare in the *Bacillus* genomes, a two-gene, type III PKS appeared widely distributed (Fig. 4A, clusters 11, 12, 15, 16, 17, 18, 22, 27, 36, 37, 38, 40, 41, and 42). The structure of this compound was first suggested in 2009 based on homologous overexpression of the two biosynthetic genes *bpsA* and *bpsB* (40). This approach yielded a number of triketide pyrones as well as small amounts of tetraketide alkylresorcinols with various chain lengths, but no native expression could be detected (40). Similar molecules isolated from *Azotobacter vinelandii* and *Streptomyces griseus* have been implicated in cyst formation and beta-lactam resistance, respectively, but no function could be ascribed to these putative alkylpyrones from *B. subtilis* (41, 42). The *bpsA-bpsB* operon is particularly well distributed among *subtilis* group strains, being found in *Bacillus amyloliquefaciens*, *Bacillus atrophaeus*, *Bacillus flexus*, *Bacillus subtilis*, and *Bacillus megaterium* strains. Moreover, 15% of the publically available genomes and 5% of the AgBiome genomes possess this BGC. The prevalence of this operon suggested that the products might have an important biological function within the *Bacilli*. We tested this prediction, and below we ascribe, for the first time, a role for the *bpsA-bpsB* operon in affecting *Bacillus* physiology, impacting both biofilm formation and sporulation.

### Exceptions and unique singletons.

Given the limited number of natural products that have been isolated from *Bacillus*, we identified a remarkably large number of unknown BGCs in the data set. The majority of these unknown BGCs comprise unique singletons that did not cluster with any other BGC; some were so rare that they did not connect with the rest of the network map. These highly unusual BGCs are potentially promising leads for discovering new compounds and biological activities.

One unexpected rare find in the *Bacillus* genomes was a small set of type II PKS BGCs. Although some polyketides have been isolated from *Bacilli*, they are predominantly of the extended, linear form, common to type I and III PKSs. To date, there have been few reports of the polyaromatic type II polyketides outside the *Actinomycetes* and none from the *Bacillales*. Remarkably, we identified four unique type II PKSs within *Bacillus* genomes from the AgBiome strains (two *B. cereus* strains, one *B. megaterium* strain, and one *B. mycoides* strain). The two easily identifiable PKS genes in these BGCs (the two ketosynthases) show approximately 45% homology to BGCs in a parcubacterial strain, the cyanobacterium *Gloeobacter violaceus*, and a number of *Delftia* species. These BGCs contain, in addition to the two ketosynthase genes, multiple genes responsible for other structural modifications. The metabolites produced by these BGCs are completely unknown, and it is likely that the presence of these BGCs in these *Bacillus* genomes is due to HGT.

**FIG 5:**
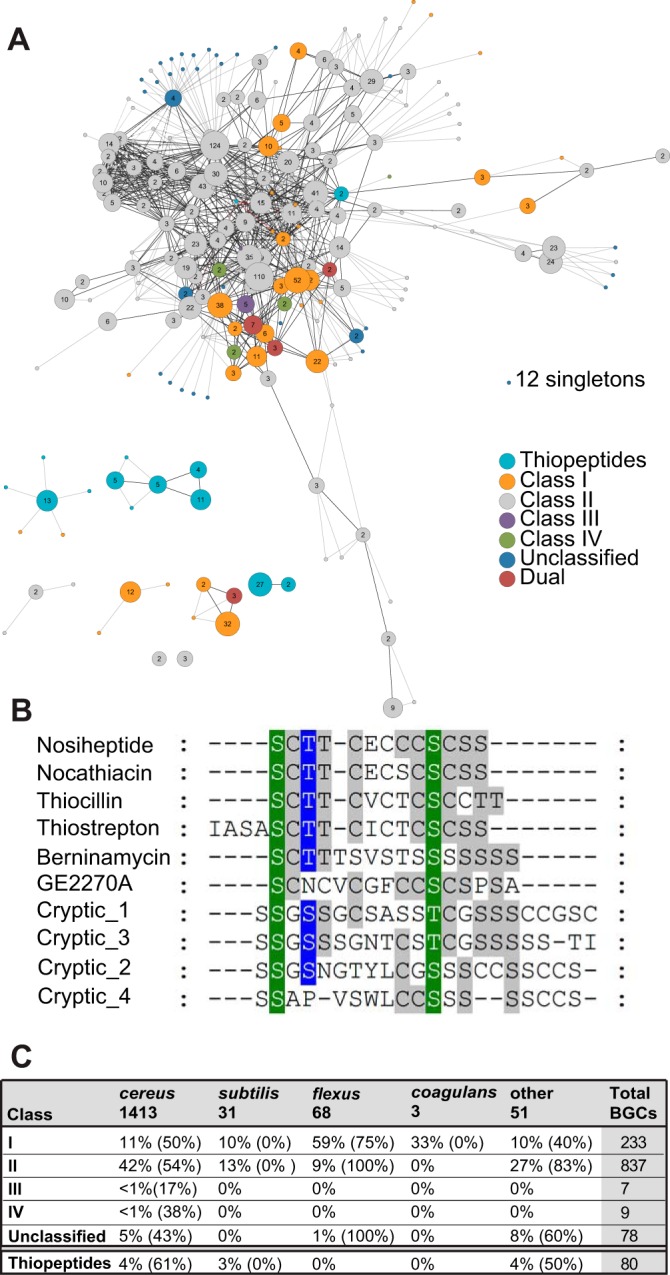
Examples of lanthipeptide and thiopeptide RiPPs. (A) Network map for lanthipeptides and thiopeptides, with the number of BGCs at each node. Colors indicate the lanthipeptide class, thiopeptide, or the lack of a class. Dual BGCs have machinery for more than one class. The number of singletons is listed. (B) Alignment of thiopeptide precursor peptide sequences showing sequence diversity from known thiopeptides from streptomycetes. (C) Distribution of lanthipeptides by class for the various species groups showing the percentage of strains containing that class of RiPP, with percentages of strains above a 75 HGT score in parentheses.

Lanthipeptides and thiopeptides in particular represented a disproportionate number of the weakly conserved BGCs that are predicted to generate new or novel compounds. Lanthipeptides are characterized by lanthionine or methyllanthionine bridges formed by a thioether linkage between two alanine residues (43, 44). In *Streptomyces*, SapB, the canonical lanthipeptide, plays a critical role in the development of aerial mycelia (45). Similarly, lanthipeptides may have important signaling roles in *Bacilli*. Lanthipeptides proved ubiquitous in *Bacillus* genomes. We identified lanthipeptide BGCs in 45% of public strains (100 genomes) and 79% of AgBiome strains (1,064 genomes) (Fig. 5A), but they are highly understudied; only five of these BGCs are associated with a known natural product. Most of these BGCs are class I and class II lanthipeptides from the *B. cereus* group, although at least one lanthipeptide can be found in every phylogenetic group (Fig. 5C). Additionally, our analysis predicts a few of the substantially more rare class III and IV lanthipeptides, as well as some severe outliers with unique combinations of multiple small type II enzymes. A few lanthipeptides produced by *Bacilli* have been studied for their antibiotic activity against Gram-positive organisms; the ceredins, lanthipeptides from the *cereus* group, are hypothesized to be produced during competence in late exponential phase, and haloduracin, a lanthipeptide produced by *B. halodurans*, can inhibit *B. anthracis* spore outgrowth (46). Little else is understood about the roles of this metabolite class in *Bacilli*, but their prominence suggests a significant role in the genus.

Thiopeptides, or thiazole-containing peptides, are macrocyclic peptides containing thiazoles; they are cyclized through a nitrogenous ring that can adopt a variety of oxidation states and often contain dehydrated residues (43). Thiopeptides have historically been found in streptomycete species; only one thiopeptide (thiocillin) is known to be produced by a *Bacillus* species (*B. cereus* ATCC 14579) and was recently found to play a role in *Bacillus subtilis* biofilm induction. We identified a total of 80 new thiopeptide BGCs in these *Bacillus* genomes. Many of these *Bacillus* thiopeptides possessed unique and remarkably diverse precursor peptide sequences relative to those present in previously characterized thiopeptides (Fig. 5B). It will be of interest to see if these new thiopeptides play roles similar to those of the thiocillins from *B. cereus* ATCC 14579.

#### Activity of *bpsA-bpsB* operon products.

Many of the BGCs identified in our extensive *Bacillus* genome database are unknown, while most of the highly prevalent and conserved BGCs have already been shown to have important roles impacting bacterial physiology. We hypothesized that other conserved but uncharacterized BGCs might also act as cell-cell signals and have physiological effects on *Bacillus*. Thus, we examined the *bpsA-bpsB* operon from the most common PKS BGC for potential biological activity.

**FIG 6:**
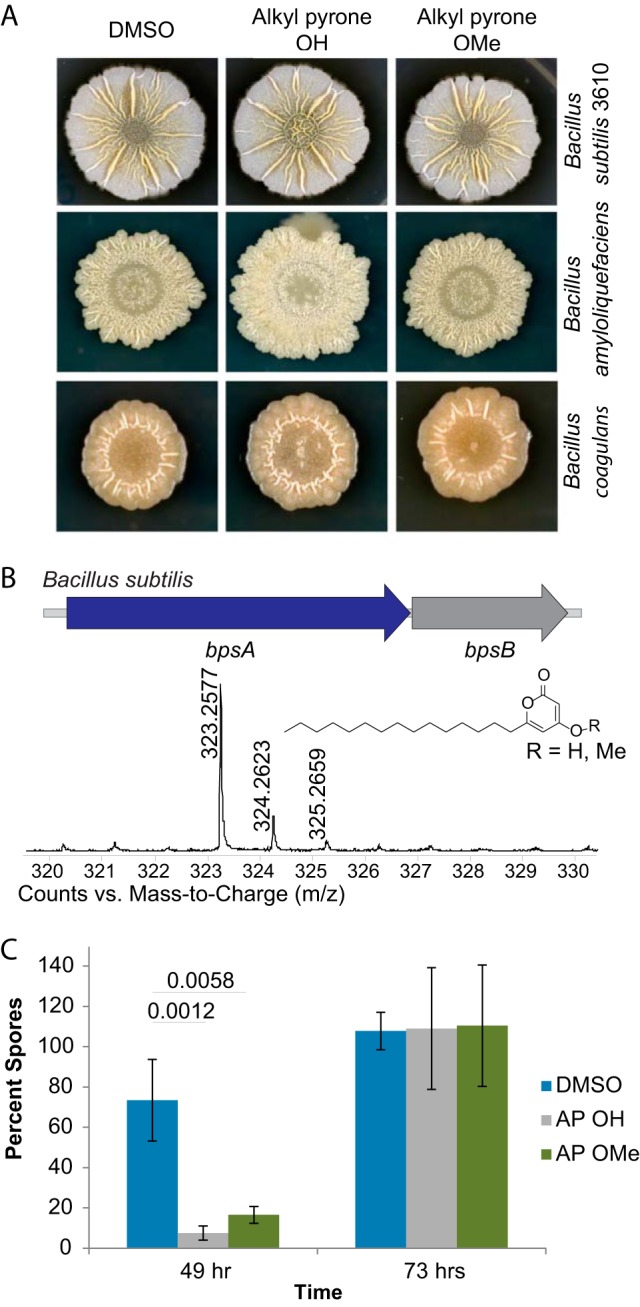
Alkylpyrone biological activity. (A) Phenotypic changes in *Bacilli* in response to alkylpyrones. All three strains show increased wrinkling in the center when treated with alkylpyrone OH. (B) Production of alkylpyrone OH in *B. subtilis* with BGC and its structure. (C) Alkylpyrones delay sporulation in *Bacillus subtilis* at 49 h. Seven samples were taken, and the error bars show standard deviations.

Products of the *bpsA-bpsB* operon have never been isolated from wild-type strains. Moreover, efforts to ascribe biological function to putative compounds from engineered strains have not been able to uncover biological activity. Two of the best-studied phenotypes of *Bacillus* species are their ability to form distinctive biofilms on solid agar (47) and their ability to sporulate; these two processes are often coregulated (12). When we examined the SubtiExpress database (48) for clues about the potential biological function of this operon, we observed that transcription of *bpsA* (the type III PKS in the BGC) is upregulated during sporulation. We therefore grew *B. subtilis* 3610, a model *Bacillus* strain, in MSgg, a biofilm-inducing medium, for 72 h, in order to allow *B. subtilis* to sporulate (47). LCMS analysis showed production of alkylpyrones with C_15_ side chains in wild-type *B. subtilis* (Fig. 6B). To further probe the biological activities of these alkylpyrones, we synthesized two variants that we identified from *B. subtilis*; both had straight C_15_ chains, but one was methylated at the 4-hydroxy position (Fig. 6B and Fig. S3).

10.1128/mSystems.00040-17.3 FIG S3  Alkylpyrone synthesis. Alkylpyrone synthesis scheme and characterization of alkylpyrone OH (A) and alkylpyrone OMe (B), including UV, high-resolution LCMS, and proton NMR results. Download FIG S3, EPS file, 1.9 MB. Copyright © 2017 Grubbs et al. 2017 Grubbs et al. This content is distributed under the terms of the Creative Commons Attribution 4.0 International license.

To discover the activities of these alkylpyrones, we screened a panel of *Bacilli* comprised of representative members from 13 different species in the presence of either one of the two purified alkylpyrones or a dimethyl sulfoxide (DMSO) solvent control when the organisms were grown on two different media (Luria-Bertani broth [LB] and MSgg) (Fig. 6A). No effect could be seen in growth or colony morphology in the presence of the unmethylated alkylpyrone. However, the 4-hydroxyl alkylpyrone affected the biofilm colony morphology of *B. subtilis* 3610 on MSgg and those of *B. amyloliquefaciens* and *B. coagulans* on LB; in all cases, we observed a hyper-wrinkling in the center of the colonies as well as other, more-subtle morphological changes (Fig. 6A). It has previously been reported that hyper-wrinkling in *B. subtilis* biofilms is due to increased cell death (49), but our data indicate that the 4-hydroxyl alkylpyrone does not impact the growth of these three strains; it does kill two other *Bacillus* strains (Fig. S4). Whether the observed effects are due to localized cell death or not, these data indicate that 4-hydroxyl alkylpyrone functions as a signal that impacts biofilm colony morphology within multiple *Bacillus* species.

10.1128/mSystems.00040-17.4 FIG S4  Alkylpyrones do not affect growth. (A) Growth curve of alkylpyrone OH-treated samples; (B) full growth curve of the three *Bacilli* with strong phenotypes; (C) total number of CFU taken during spore counts with 6 samples. Download FIG S4, EPS file, 1.2 MB. Copyright © 2017 Grubbs et al. 2017 Grubbs et al. This content is distributed under the terms of the Creative Commons Attribution 4.0 International license.

Given the timing of *bpsA* expression in *B. subtilis* and the regulatory relationship between biofilm formation and sporulation, we also sought to investigate a potential role for these alkylpyrones in sporulation. We tested effects of both alkylpyrone variants on *B. subtilis* sporulation by adding compound or DMSO as a control to cells in liquid MSgg and monitoring the percentage of spores formed over time. Both alkylpyrones delayed sporulation at day 2 compared to sporulation in the DMSO control (Fig. 6C) at concentrations that did not affect growth (Fig. S4). However, by day 3, the percentage of spores formed in the alkylpyrone-treated sample was comparable to the percentage of spores observed in the DMSO control (Fig. S4).

The *Bacillus* alkylpyrones have structural similarity to the germicidins from streptomycetes in that they have the same 2-pyrone core but differing alkyl substituents; germicidins are known to delay the germination of streptomycete spores (50, 51). We therefore sought to assess whether the alkylpyrones might also impact the germination of either *Bacillus* or *Streptomyces* spores. We tested this by counting the CFU resulting from spreading *B. subtilis* spores on rich medium containing the alkylpyrones and monitoring *S. coelicolor* germination in fresh liquid medium by absorbance determination and phase-contrast microscopy (52). In neither case did we see any evidence of the alkylpyrones affecting germination (Fig. S5). Thus, like the germicidins, alkylpyrones seem to regulate sporulation, but they do so by delaying sporulation rather than by impacting germination.

10.1128/mSystems.00040-17.5 FIG S5  Alkylpyrones do not affect germination. (A and B) Germination assay for *Bacillus subtilis* (A) and *Streptomyces coelicolor* (B) spores treated with alkylpyrones. Images were taken at 6 h. Absorbance and numbers of CFU were measured with 3 samples each. Download FIG S5, PDF file, 1.2 MB. Copyright © 2017 Grubbs et al. 2017 Grubbs et al. This content is distributed under the terms of the Creative Commons Attribution 4.0 International license.

## DISCUSSION

Here we have pursued an extensive bioinformatics analysis of the genus *Bacillus* in order to measure its chemical diversity and better understand the role of its specialized metabolites. One of the unique features of our study is the substantial number of genome sequences from a single genus included in our analysis. By examining 1,566 genomes, we have evaluated nearly five times more sequences than the next-most-extensive published analysis of the *Bacillus* genus (20). In addition, more than 85% of these genomes are being made newly available to the public (the other 15% are already publicly available), greatly enhancing the available database of *Bacillus* genome sequences. Although the phylogenetic diversity of our genomic database is biased toward the *cereus* group, this also provided us with the opportunity to more deeply sample this important portion of the *Bacillus* taxon. One important question is whether this enlarged genome database provided any informational value beyond what would have been attained using the previously available 221 public genomes. In fact, a number of our conclusions were possible only as a result the inclusion of these additional AgBiome genomes, including (i) the widespread prevalence of the CATCATR BGCs, (ii) the existence of type II PKS singletons in *Bacilli* (seen only in the AgBiome genomes), and (iii) the diversity of the thiopeptides (seven of the thiocillin DBSCAN clusters, comprised of 67 BGCs, were present only in the AgBiome genomes). These results argue, consistently with previous studies (53, 54), that expanding our knowledge of the genomic content of additional bacterial strains will continue to reveal new insights into natural product diversity and function.

The next-most-extensive analysis of the genus *Bacillus*, using 328 sequenced *Bacillales* available from the NCBI database, arrived at results similar to ours (20). Those researchers utilized a related workflow in that they used antiSMASH to identify BGCs and then BLAST (but not MultiGeneBlast) to compare the identified BGCs, although BAGEL3 was also used to identify BGCs (20). However, they did no further clustering analysis to better characterize or visualize the biosynthetic diversity present among *Bacillus* BGCs and provided no experimental validation. Their strain set was more diverse than ours but provided less depth in terms of the number of strains for each species (20). Their analysis focused primarily on the RiPPs, and they found BGCs for 105 lanthipeptides and 5 thiopeptides (20). In comparison, our greater number of genomes afforded 1,164 lanthipeptides and 80 thiopeptides. Their analysis uncovered 10 novel NRPS, PKS, and hybrid BGCs (20), which agrees with our conclusions that many of these important compounds are known and well conserved.

**FIG 7:**
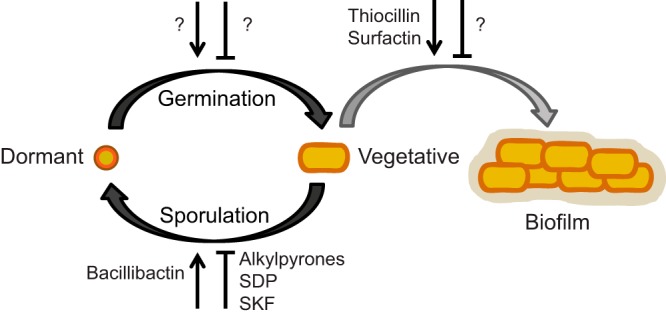
*Bacillus* life cycle highlighting the signaling-specialized metabolites that mediate each step. Compounds are noted as promoting (arrow) or delaying (flat-ended symbol), and question marks indicate where no metabolite has yet been assigned a signaling role.

Since *B. subtilis* was first described by Ferdinand Cohn in the late 1800s, it has been known to have a complex developmental life cycle (55). Some of this cellular differentiation occurs in response to self-produced specialized metabolites (12). Signaling-specialized metabolites that impact the *Bacillus* life cycle, including the newly characterized alkylpyrones, are summarized in Fig. 7. Our analysis reveals that many of the biosynthetic pathways for these metabolites are conserved across either the entire *Bacillus* genus or within specific phylogenetic clades. Biofilm formation is one of the most well-studied phenotypes exhibited by *Bacillus* (56) and confers upon these strains the ecologically important ability to adhere to plant roots, providing plants with pathogen resistance (57); many of the most highly conserved metabolites identified in our analysis have been shown to have a role in biofilm development. Specifically, the NRPS lipopeptide surfactin triggers biofilm formation in *B. subtilis* (13); we predict that this function is conserved across the entire *subtilis* group based on its extremely high conservation (97% of strains). The structurally related kurstakins have similarly been shown be important for swarming and biofilm formation in *B. cereus* (31) and are highly conserved within the *cereus* group (76% of strains). We thus hypothesize that the contribution of the kurstakins to these phenotypes may account for its high prevalence in the *cereus* group, while surfactin fulfills these functions among the *subtilis* group strains.

Such separation of natural product conservation along phylogenetic lines appears common. For instance, other prevalent, conserved compounds of known functions (such as bacitracin, cereulide, and zwittermicin) were each observed only within the *cereus* or “other” groups, while fengycin, bacillaene, iturin, and difficidin were essentially observed only within the *subtilis* group. This suggests that the distinct environmental niches that members of the *cereus* and *subtilis* clades inhabit may select for the conservation of metabolites with distinct (or potentially redundant) beneficial functions. Indeed, characterizing the metabolic coding signature of individual strains may represent an environmentally relevant mechanism to classify strains by their functional ecotypes (58, 59). Interestingly, as also noted in reference 20, we see that NRPSs appear more frequently among *cereus* group members but that PKS and NRPS-PKS hybrids appear more frequently among *subtilis* group members. This phylogenetic division is likewise detected among natural product BGCs of unknown functions; bacillamide is present in approximately a third of the *cereus* group members, and the CATCATR metabolite BGC is made by approximately a quarter of them. Although these metabolites still have completely unknown functions, we hypothesize that their prominence among these strains indicates that they may serve important biological roles within the producing species.

We sought to validate this hypothesis by examining another highly conserved metabolite from our analysis, the *bpsA-bpsB* alkylpyrone. Although an engineered strain had provided partial evidence of the structure of this compound, it could not be induced in its native host, and no biological function could be ascribed to the putative structures. Based on published expression data, we found conditions that elicited production of the native alkylpyrone BGC in *B. subtilis*. We further hypothesized that the alkylpyrones might potentially have a role in sporulation and biofilm development. Indeed, using synthetic material, we demonstrated that alkylpyrones delay sporulation in *B. subtilis* and that the 4-hydroxy alkylpyrone also caused phenotypic changes in the biofilm colony morphologies of multiple *Bacillus* species. Sporulation is a defining morphological feature of *Bacillus* species that is also relevant to its environmental survival; thus, it is not surprising that *Bacilli* might generate metabolites to regulate this important process. Indeed, the *Bacillus* peptide metabolite SDP (sporulation-delaying protein), was initially identified for its ability to delay sporulation in *B. subtilis* (60), although the purified compound was later found to cause cell lysis and death (61). 2,4-Diacetlyphloroglucinol (DAPG), a metabolite produced by *Pseudomonas protegens*, has also been shown to delay sporulation and biofilm formation in *B. subtilis* (62), but it similarly causes cell death at higher concentrations. Thus, the alkylpyrones are the first identified natural products that negatively impact the process of sporulation without inhibiting growth.

In addition to identifying these highly conserved BGCs, we identified a number of unique, weakly conserved BGCs across all species and BGC classes. Some of these were entirely distinct “singletons,” while others were BGCs that were conserved in only a small number of strains (e.g., the unknown NRPS BGCs, present in <5% of the *cereus* strains, and many of the RiPPs). The majority of these BGCs seem to have been acquired through horizontal gene transfer, particularly the singletons. Certain strains of *Bacilli* contained more distinct “singletons” than others; a strain of *Bacillus pseudomycoides* in particular possesses eight unknown singletons (see Fig. S6 in the supplemental material). These BGCs are quite diverse and offer avenues for identifying new and distinct natural products that appear to occupy untapped niches of chemical space. Our analysis further suggests that these metabolites may similarly exhibit previously unknown biological activities that impact the physiology and ecological interactions of the producing bacteria.

10.1128/mSystems.00040-17.6 FIG S6  *Bacillus* superproducers of distinct singleton BGCs. The graph shows the distribution of strains containing one or multiple singletons. Download FIG S6, EPS file, 0.7 MB. Copyright © 2017 Grubbs et al. 2017 Grubbs et al. This content is distributed under the terms of the Creative Commons Attribution 4.0 International license.

## MATERIALS AND METHODS

### Genome sequencing.

Liquid cultures of environmental *Bacillus* isolates were grown and spun down, and DNA was isolated from the resulting cell pellets using MoBio microbial DNA isolation kits. The resulting DNA was quantified using a Quant iT PicoGreen assay. One nanogram of quantified DNA was sheared enzymatically at 55°C for 5 min using the Illumina Nextera XT tagmentation enzyme. Tagmented DNA fragments were enriched by 10 cycles of PCR amplification using PCR master mix and primers with the index from Illumina. Libraries were quantified by the KAPA SYBR fast quantitative PCR (qPCR) kit and pooled at a 4 nM concentration. Libraries were denatured with 0.2 N NaOH and sequenced on an Illumina HiSeq sequencing platform. Illumina paired-end reads were demultiplexed using Illumina software bcl2fastq v2.18.0.12. Paired-end reads were adapter and quality trimmed using cutadapt version 1.5 as recommended by Illumina. Trimmed paired-end reads were assembled and reads were aligned back to the consensus sequence using the CLC Genomics programs CLC Assembly Cell and CLC Mapper from Qiagen.

### Species identification.

Barrnap was used to identify 16S and 23S ribosomal gene sequences in each genome. Species were assigned based on top hits in BLAST searches against the Genomic Reference Sequence Database (GenomicRefSeq).

### Biosynthetic gene cluster prediction and comparison.

The analysis pipeline is outlined in Fig. 1. antiSMASH was used with default parameters to predict BGCs present in each genome. All BGCs were then divided into individual GenBank files, and an initial pairwise similarity matrix was calculated using MGB with default parameters to produce scores, based on an algorithm unique to MGB, between every possible BGC pair. Scores between every given pair of BGCs were averaged, as they might differ depending on the direction of the comparison. This produced a “lower left” similarity matrix that was then normalized by dividing each column of values by the maximum value in the respective column. This was then converted to a distance matrix by inverting the values. Submatrices were produced for each type of BGC based on antiSMASH assignment.

### Clustering of biosynthetic gene clusters.

The DBSCAN implementation available in the R fpc library was used in conjunction with the previously produced submatrices to cluster BGCs. DBSCAN uses two key parameters for its algorithm: EPS and min pts. DBSCAN clusters the BGCs by their MultiGeneBlast scores if a certain number of BGCs (min pts) fall within a certain radius (EPS). DBSCAN parameters were optimized by iteratively running DBSCAN and varying the EPS by 0.01 (from 0.01 to 0.98) and MinPts by 1 (from 2 to 15) in every combination. The resulting cluster listings were compared to the graphic alignments from MultiGeneBlast. In most cases, MGB alignments would quickly deteriorate, and those DBSCAN parameters that best discriminated at these borders were chosen. Those alignments corresponding to known BGCs were most heavily considered in DBSCAN parameter optimization. See Table S2 in the supplemental material for final parameters.

### Network visualization.

Cytoscape (25) was used to build network maps that allowed the relationships between the DBSCAN clusters to be visualized.

### Horizontal gene transfer analysis.

Alien_hunter (26) was used to predict DNA regions that are likely the result of HGT. These results were used to calculate the percentage of each BGC that was obtained via HGT.

### Custom scripts.

Scripts were used to convert formats between programs and facilitate the automation of running DBSCAN over the large number of parameters tested. All analyses and computations were completed using the programs cited.

### Silhouette coefficient calculations.

The silhouette coefficient is calculated using the mean intracluster distance (*a*) and the mean nearest-cluster distance (*b*) for each sample. The silhouette coefficient for a sample is (*b* − *a*)/maximum (*a*, *b*) (24). To clarify, *b* is the distance between a sample and the nearest cluster of which the sample is not a part. See Table S2 for calculated silhouette coefficients for each BGC class.

### Strains.

For the list of strains used in the phenotypic screens, see Table S3. All strains tested for phenotypes and other public strains were from our laboratory collection. The nonpublic strains used in the LCMS analysis came from AgBiome.

10.1128/mSystems.00040-17.9 TABLE S3  Strains tested in the phenotypic screen. Download TABLE S3, EPS file, 0.8 MB. Copyright © 2017 Grubbs et al. 2017 Grubbs et al. This content is distributed under the terms of the Creative Commons Attribution 4.0 International license.

### Growth curves.

All strains used in the phenotypic screen were resuspended in Luria-Bertani broth (LB)-Lennox (10 g tryptone, 5 g yeast extract, and 5 g NaCl per liter) to an optical density at 600 nm (OD_600_) of 0.05, and 150 µl was transferred to a 96-well plate. Compound was added at 50 µM in DMSO. The plate was shaken at 30°C and 350 rpm, while absorbance measurements were taken on an Infinite m200 Pro Tecan.

### Phenotypic screens.

The alkylpyrone compounds were tested against 13 strains of *Bacilli* (Table S3) on Lennox-LB and MSgg (5 mM potassium phosphate [pH 7], 100 mM morpholinepropanesulfonic acid [MOPS; pH 7], 2 mM MgCl_2_, 700 μM CaCl_2_, 50 μM MnCl_2_, 50 μM FeCl_3_, 1 μM ZnCl_2_, 2 μM thiamine, 0.5% glycerol, 0.5% glutamate) with 1.5% (wt/vol) agar. Plates were poured with a Wheaton Unispense liquid dispenser into 20 ml for LB or 30 ml for MSgg. All liquid medium was the same composition but without the agar. *S. coelicolor* was grown on soy flour-mannitol (SFM) agar (2% soy flour, 2% mannitol, 1.5% agar) or in glucose-yeast extract-malt extract (GYM) liquid medium (0.4% glucose, 0.4% yeast extract, 1% malt extract). Plates were first spotted with 2.5 µl of a 100 µM concentration of a compound, and then 1 µl of cells at an OD_600_ of 0.5 was spotted on top of the dried compound. Plates were incubated at 30°C, and photos were taken at 72 h. The images shown are of the strongest phenotypes across all combinations and were representative of three biological replicates.

### Spore counts.

Spore counts were taken for *B. subtilis* 3610 in the presence of 50 µM compound. *Bacillus subtilis* was grown in 3 ml of LB at 37°C to an OD_600_ of 1.0. The culture was diluted 0.2 µl in 1 ml of MSgg broth, and compound was added into DMSO to 50 µM before being allowed to grow at 30°C and 200 rpm. At each time point, 100 µl of culture was sonicated using a protocol verified to separate cells from one another without lysing them (63) (on a Branson digital sonifier for 12 s and 15% amplitude, with a pulse of 1 s on and 1 s off). Half of the sample was set aside, and the other half was heated at 80°C for 30 min. The samples were serially diluted in LB and grown on LB plates for colony counting the next day using 6 biological replicates.

### *Bacillus subtilis* germination assay.

*B. subtilis* was grown under the same conditions as for spore counts. At 49 h, the sample was sonicated, heat treated to kill vegetative cells, and serially diluted using the same method as for spore counts. The sample was grown on LB plates containing a 50 µM concentration of either compound or DMSO to count the colonies that would germinate in the presence of compound.

### *Streptomyces coelicolor* spore isolation.

*S. coelicolor* was grown on SFM agar for 5 days at 30°C until the plate was covered in tan spores. Spores were gently scraped off the plate using an inoculating loop and resuspended in 5 ml of water. The spores were washed 3 times by pelleting and resuspending them in 3 ml water before filtration through Miracloth to remove any mycelia. Spores were heated at 50°C for 10 min to kill any vegetative cells and stored in water at 4°C.

### *Streptomyces coelicolor* germination assay.

Absorbance measurements for germination were done by diluting spores 1:10 in GYM medium in a 96-well plate with 50 µM compound in DMSO. The plate was shaken at 30°C and 300 rpm, while absorbance measurements were taken on an Infinite m200 Pro Tecan. The assay was run in triplicate.

### Microscopy.

At each time point, 5 µl of germinating spores was spotted on a 1% agar pad on a glass slide and covered with a coverslip. Images were acquired on a Nikon Eclipse 80i microscope with a 40× objective.

### Statistics.

The *P* values were calculated with Tukey’s honestly significant difference test using JMP Pro 12 software.

### Growth conditions and extractions for LCMS evaluation.

All AgBiome strains for kurstakin production were grown in 3 ml of LB at 30°C in a roller drum incubator for 72 h. Cells were pelleted at 4,000 rpm, resuspended in 0.5 ml of methanol, and allowed to sit at least 1 h for extraction. Cells were pelleted again at 15,000 rpm, and the resulting methanol supernatant was diluted 1:10 in 1:1 acetonitrile-water for injection on the LCMS. All AgBiome strains for bacillamide production and public strains were grown in 50 ml of LB at 30°C and 200 rpm for 30 h. For alkylpyrone production, *B. subtilis* was grown in 50 ml of MSgg at 30°C and 200 rpm for 72 h. For extraction, the cells were pelleted at 4,000 rpm for 25 min and sonicated using a Branson digital sonifier for 20 s at 30% amplitude with a pulse of 0.5 s on and 1.5 s off. The pH was adjusted to 2 using HCl before extraction with 1 ml of ethyl acetate for at least 1 h. Cells were pelleted again at 15,000 rpm for 5 min, and the ethyl acetate was diluted 1:10 in 1:1 acetonitrile-water for injection on the LCMS.

### LCMS.

All LCMS data were obtained on an Agilent 6520 accurate-mass quadrupole time of flight (Q-TOF) mass spectrometer with an electrospray ionization source in positive-ion mode. A 250-V fragmentor voltage was used with a 350°C drying gas temperature. The extracts were run on a reverse-phase Kinetex column using the following method: acetonitrile with 0.1% formic acid was run as a gradient from 2% to 100% over 15 min and held at 100% for 2 min with water containing 0.1% formic acid.

### Synthesis.

Alkylpyrones OH and methoxy (OMe) were synthesized according to procedures in references 64 to 66. All reactions were carried out in an oven-dried, round-bottom flask with stirring as noted in Fig. S3. All purifications were done through flash chromatography. Nuclear magnetic resonance (NMR) spectra were taken with a Varian Inova 400 (400-MHz and 100-MHz, respectively) spectrometer.

### Data availability.

Strain names, species, accession numbers, environmental collection metadata, and BGC information are all contained in Data Set S1 in the supplemental material. All genomes are available in the NCBI database under accession numbers NUFI00000000 to NVPI00000000. These whole-genome shotgun project results have been deposited in DDBJ, ENA, and GenBank under the accession number XXXX00000000, where the XXXX is replaced with the correct strain designator as indicated in Data Set S1. The versions described in this paper are version XXXX01000000, where the XXXX is replaced with the correct strain designator as indicated in Data Set S1. Scripts used to perform analyses are available at the Carolina Digital Repository at https://doi.org/10.17615/C6HT0Q.

10.1128/mSystems.00040-17.10 DATA SET S1  All identified *Bacillus* BGCs and strain metadata. Download DATA SET S1, XLSX file, 0.6 MB. Copyright © 2017 Grubbs et al. 2017 Grubbs et al. This content is distributed under the terms of the Creative Commons Attribution 4.0 International license.
