# Supplementary figures and images for: Large-Scale Bioinformatics Analysis of Bacillus Genomes Uncovers Conserved Roles of Natural Products in Bacterial Physiology
Source: mSystems. 2017 Nov 14;2(6):e00040-17. doi: 10.1128/mSystems.00040-17 (PMC5686519; doi:10.1128/mSystems.00040-17)

**A**

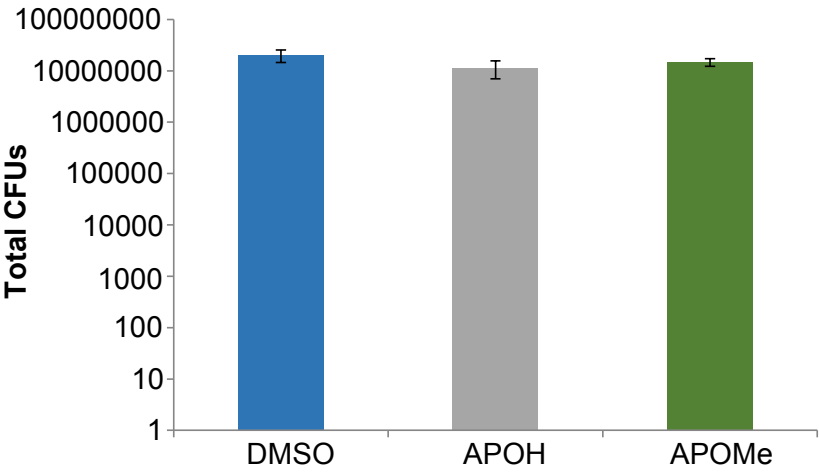

**B**

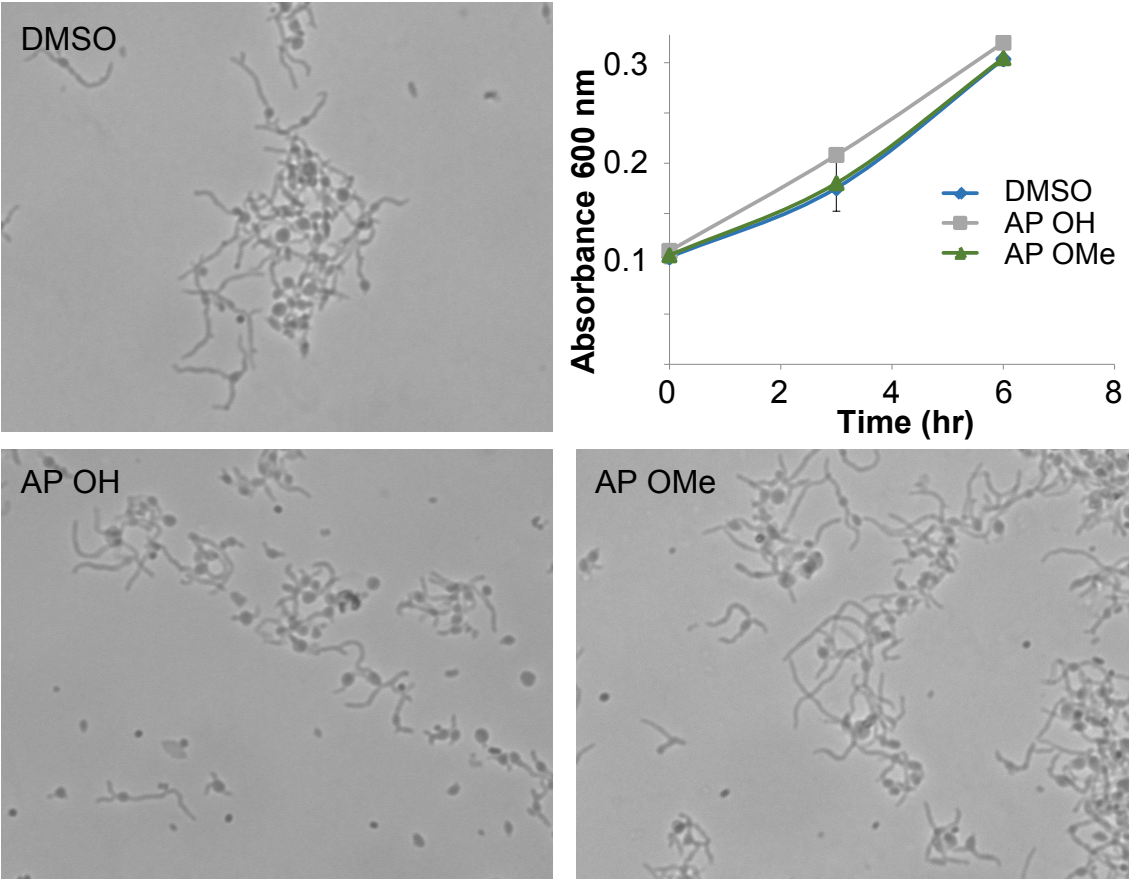

Supplement: FIG S5 [file sys001162146sf5.pdf]
